# Extracellular tau stimulates phagocytosis of living neurons by activated microglia via Toll-like 4 receptor–NLRP3 inflammasome–caspase-1 signalling axis

**DOI:** 10.1038/s41598-023-37887-3

**Published:** 2023-07-04

**Authors:** Katryna Pampuscenko, Ramune Morkuniene, Lukas Krasauskas, Vytautas Smirnovas, Guy C. Brown, Vilmante Borutaite

**Affiliations:** 1 Neuroscience Institute, Lithuanian University of Health Sciences, 50161 Kaunas, Lithuania; 2 Life Sciences Center, Institute of Biotechnology, Vilnius University, 10257 Vilnius, Lithuania; 3 Department of Biochemistry, University of Cambridge, Cambridge, CB2 1QW UK

**Keywords:** Dementia, Neurodegeneration, Cell death, Mechanisms of disease

## Abstract

In tauopathies, abnormal deposition of intracellular tau protein followed by gradual elevation of tau in cerebrospinal fluids and neuronal loss has been documented, however, the mechanism how actually neurons die under tau pathology is largely unknown. We have previously shown that extracellular tau protein (2N4R isoform) can stimulate microglia to phagocytose live neurons, i.e. cause neuronal death by primary phagocytosis, also known as phagoptosis. Here we show that tau protein induced caspase-1 activation in microglial cells via ‘Toll-like’ 4 (TLR4) receptors and neutral sphingomyelinase. Tau-induced neuronal loss was blocked by caspase-1 inhibitors (Ac-YVAD-CHO and VX-765) as well as by TLR4 antibodies. Inhibition of caspase-1 by Ac-YVAD-CHO prevented tau-induced exposure of phosphatidylserine on the outer leaflet of neuronal membranes and reduced microglial phagocytic activity. We also show that suppression of NLRP3 inflammasome, which is down-stream of TLR4 receptors and mediates caspase-1 activation, by a specific inhibitor (MCC550) also prevented tau-induced neuronal loss. Moreover, NADPH oxidase is also involved in tau-induced neurotoxicity since neuronal loss was abolished by its pharmacological inhibitor. Overall, our data indicate that extracellular tau protein stimulates microglia to phagocytose live neurons via Toll-like 4 receptor–NLRP3 inflammasome–caspase-1 axis and NADPH oxidase, each of which may serve as a potential molecular target for pharmacological treatment of tauopathies.

## Introduction

Tauopathies are a group of heterogeneous neurodegenerative diseases, characterized by tau (tubulin-associated unit) protein deposition^1^. Progressive neuronal loss and brain atrophy are prominent pathological features of these diseases, underlying deterioration of cognitive and motor functions^2^. Tauopathies affect millions of people worldwide, but the current therapies are symptomatic and do not prevent the disease progression^3^.

Normally, tau protein is a mainly monomeric axonal protein, which binds to tubulin heterodimers and hereby stabilizes microtubules. However, under pathological conditions various post-translational modifications or mutations promote tau protein detachment and mislocalization leading to accumulation of filamentous tau structures^4^. Although insoluble intracellular tau inclusions are the predominant histopathological feature correlating with the progression of cognitive decline and other clinical symptoms, they are not considered as a sole cause of neuronal death^5–7^. Various tau species also accumulate in extracellular compartments (cerebrospinal and interstitial fluid)^8^, and emerging experimental evidence suggests that tau-pathology spreads from cell to cell in a prion-like manner^9^. Extracellular tau is capable of inducing memory impairment, synaptic damage and neuronal loss^10–14^, but the exact mechanisms underlying neuronal cell death induced by extracellular tau are not well understood.

Tauopathies are also characterized by neuroinflammatory processes such as microgliosis, astrogliosis, oxidative stress and increased levels of pro-inflammatory cytokines^15,16^. In post-mortem brains from Alzheimer’s disease (AD) and frontotemporal dementia (FTD) patients, activated microglial cells have been found near neurons containing tau-inclusions^17,18^. Moreover, distinct tau inclusions have been observed within glial cells (astrocytes and oligodendrocytes)^19^, and microglia from AD brains were shown to contain tau species that normally are not found in these cells^20^. In tau transgenic mice, microglial activation is an early event preceding formation of insoluble neuronal inclusions^21,22^. In response to tau pathology, microglia display morphological and dynamical changes, secrete pro-inflammatory factors and become highly phagocytic^23–27^. Moreover, anti-inflammatory compounds and overexpression of fractalkine have been shown to suppress neurodegenerative processes in several models of tauopathies^21,28–32^. Furthermore, microglial complement components and TREM2 receptors have been found to be involved in synaptic and neuronal loss in several tau transgenic mouse models^33–36^. Importantly, genome-wide association studies link the risk for AD with innate immunity genes, including microglial phagocytic receptors CD33, TREM2, CR1 etc.^37,38^. These findings suggest that microglial activation plays a crucial role in tau-related pathologies.

In adult healthy brain, microglia protects neuronal cells by removing dead cells and pathogens. However, disruption of microglial homeostasis can lead to aberrant phagocytosis of stressed-but-viable neurons, resulting in death of the engulfed neurons—a type of cell death called primary phagocytosis or phagoptosis^39^. Death by phagoptosis was found to occur in rat brain cell cultures after exposure to UDP, lipopolysaccharide (LPS) or β-amyloid (Aβ), and in vivo after LPS or Aβ injection into rat/mouse striatum, and after transient focal ischemia in mouse brain^40–42^. Microglia were also found to phagocytose viable dopaminergic and GABAergic neurons in a transgenic *Drosophila* model, and photoreceptor cells in a retinal degeneration mouse model^43^. Experimental evidence suggests that phagocytosis also contributes to neuronal loss under tau pathology. Recently, we have shown that extracellular monomeric and pre-aggregated tau protein stimulates microglial phagocytosis of living neurons in neuronal-glial co-cultures^44^. Neurons containing P301S tau inclusions have been also shown to die as a result of being phagocytosed alive by microglia in vitro^45^. In addition, knockout of the P2Y6 receptor, required for microglial phagocytosis of neurons, prevented loss of neurons in tau treated cell cultures, and prevented both neuronal loss and behavioural deficits in a P301S tau mice model^46^. Thus, there is experimental evidence of tau-induced neuronal loss, but the precise molecular mechanisms underlying this neuronal loss are not well understood. Understanding these mechanisms could help devise new therapies to prevent this loss in tauopathies.

Microglial cells express several types of receptors inducing microglial activation^47^. Toll-like 4 (TLR4) receptors are transmembrane proteins that can be activated by different pathogen-associated molecular patterns (PAMPs) as well as amyloidogenic proteins^48–51^. TLR4 signalling can trigger activation of caspase-1, an inflammatory cysteine protease, activation of which is associated with the neuroinflammation observed in AD and other neurodegenerative disorders^49,52–55^. Caspase-1 itself becomes activated by cytosolic multi-protein complexes called inflammasomes^53^. Recently, extracellular and intracellular tau proteins were demonstrated to activate microglial cells via the NLRP3 inflammasome^26^, mediating tau pathology, spreading and brain atrophy in P301S tau mice model^56,57^. TLR4 signalling is also known to promote activation of the NADPH oxidase (NOX), which is an important source of reactive oxygen species (ROS)^58,59^, and which along with TLR4 receptors were found to be implicated in Aβ induced microglial phagocytosis of neurons^41^. Therefore, in this study, we aimed to investigate whether TLR4 receptors, NLRP3 inflammasome, caspase-1 and NOX are involved in extracellular tau- induced neuronal loss.

## Materials and methods

### Materials and reagents

Alexa Fluor™ 488 Protein Labelling Kit, *E. coli* BL21 Star™ (DE3) strain, isolectin GS-IB_4_ from *Griffonia simplicifolia* conjugated with Alexa Fluor488 or Alexa Fluor568, IL-18 (Rat) ELISA kit, pHrodo™ red *E.coli* BioParticles conjugate, were purchased from *Invitrogen, ThermoFisher Scientific* (USA). Cell culture reagents DMEM Glutamax, fetal bovine serum, horse serum, penicillin–streptomycin, Versene (1:5000) solution were from *Gibco, ThermoFisher Scientific* (USA). Poly-(l)-lysine was from R&D systems (USA), IL-1β (Rat) ELISA kit from *Abbexa* (United Kingdom), FamFlica Caspase-1 Assay Kit from *ImmunoChemistry Technologies* (USA), anti-TLR4 antibody from *Santa Cruz Biotechnology* (USA). Cell permeable Ac-YVAD-CHO, VX-765, GSK2795039, MCC950, and IgG were obtained from *Merck* (Germany). All other materials were purchased from *Sigma-Aldrich* (USA).

### Recombinant tau protein expression

Recombinant human tau protein (2N4R isoform) was expressed in *E. coli* BL21 Star™ (DE3) strain and purified as described previously^60^. Briefly, tau protein was fused at the N-terminus with His-Sumo tag, purified using Ni^+2^ ion affinity chromatography. After His-Sumo cleavage with ULP1 protease, second affinity chromatography step was used together with size exclusion chromatography. Protein was collected, lyophilized and stored at − 20 °C.

### Cell cultures and treatments

Cell cultures were prepared from 5 to 7-days-old Wistar rats of both sexes.

**Figure 1:**
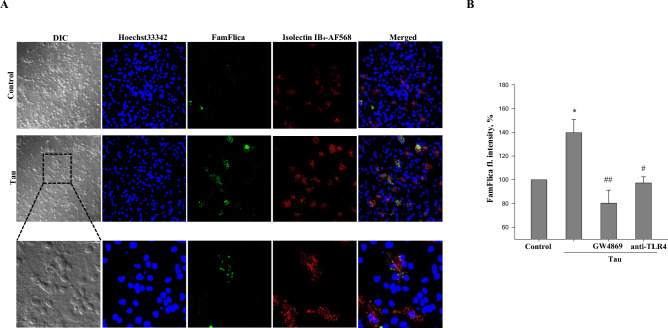
Tau induces caspase-1 activation in microglial cells via TLR4 receptors and neutral sphingomyelinase (nSMase). (**A**) representative confocal microscopy images of caspase-1 labelling in untreated (control) or treated with 3 µM tau for 24 h neuronal glial-co-cultures. Neuronal cell can be identified in differential interference contrast (DIC) images. Cell nuclei were labelled with Hoechst 33342 (blue), microglial cells with isolectin B_4_-AlexaFluor568 (red) and activated caspase-1 with FamFlica reagent (green). Scale bars, 10 µm. (**B**) FamFlica fluorescence intensity in microglial cells. Pure cell cultures were incubated with 3 µM tau for 24 h with or without 1 µg/mL anti-TLR4 antibody or 11 µM nSMase inhibitor GW4869. FamFlica fluorescence intensity in tau-treated (with/without inhibitors) groups is expressed as the percentage of fluorescence intensity in the control group (untreated), which were considered as 100%. Data are presented as means ± SE for 3 independent experiments; *p < 0.05 versus untreated control, ^#^p < 0.05, ^##^p < 0.01 versus tau treated cultures.

Neuronal-glial co-cultures were prepared from rat cerebellum as described^61^. In brief, cerebellum was dissociated in Versene (1:5000) and suspended in DMEM Glutamax growth medium supplemented with 5% fetal bovine serum (FBS), 5% horse serum, 13 mM glucose, 20 mM KCl and 1% penicillin/streptomycin (P/S). Cell suspension was plated in 1 μg/mL poly-(l)-lysine (PLL) coated 96 well plates at 0.5 × 10^6^ cells/ml density. For confocal microscopy, cell cultures were plated on 1 μg/mL PLL coated glass coverslips (24 well plates) at 10^6^ cells/ml density. Mixed cell cultures were grown for 6–7 days before treatments. These cultures consisted of 82 ± 3% neurons (Neu-N-positive), 7 ± 1% microglia (Iba-1- and isolectin IB_4_-positive) and 11 ± 1% astrocytes (GFAP-positive) (Fig. 1S).

Pure microglial cultures were prepared from rat cortices as described^44^. Briefly, after blood vessels and meninges were removed, cortices were dissociated in Versene (1:5000) solution. Cell suspension was centrifuged and re-suspended in DMEM Glutamax growth medium supplemented with 10% FBS and 0.1% P/S. Astroglial cell suspension was plated into 0.1 μg/mL PLL coated T75 flasks and grown for 7–10 days. Pure microglial cell cultures were obtained by mechanical detachment and plated in 0.1 μg/mL PLL coated 96 well plates at 10^5^ cells/mL density. Microglial cells were allowed to attach overnight before experiments. The purity of microglial cell cultures was 96 ± 2%.

For the experiments, cell cultures were pre-incubated with 1 μM Ac-YVAD-CHO, 1 μM GSK2795039, 11 μM GW4869, 1 μg/mL anti-TLR4, 1 μg/mL IgG for 30 min and with 200 nM VX-765 for 2 h before treatment with tau protein. Monomeric tau protein (2N4R isoform) was prepared as described^44^. Lyophilized tau was dissolved in 10 mM HEPES buffer at 1 mg/mL concentration, aliquoted and stored at − 80 °C. For viability and cell density assay, neuronal-glial co-cultures were treated with 3 μM (138 μg/mL, MW 45.9 kDa) tau. Endotoxin level in recombinant tau protein samples was evaluated by Pierce Chromogenic Endotoxin Quant Kit as described^44^, and was found not to exceed 0.007%.

### Cell viability assessment

**Figure 2:**
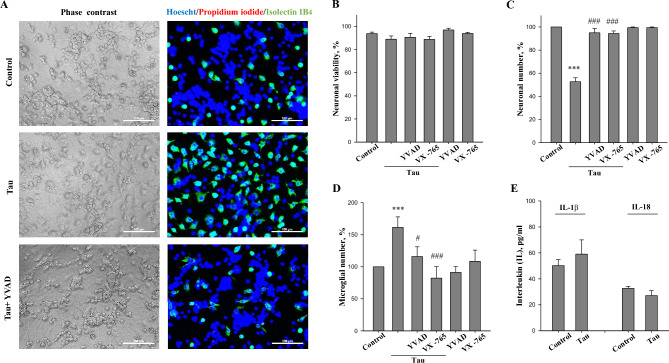
Tau-induced neurotoxicity is prevented by caspase-1 inhibitors. (**A**) representative images of untreated (control) and tau treated (3 µM for 48 h) neuronal glial-co-cultures with or without 1 µM YVAD-CHO. In phase contrast images, neuronal cell can be identified by characteristic shape and morphology. Cell nuclei were labelled with Hoechst 33342: cells with homogeneously stained nuclei (blue) were considered as viable, cells with condensed or fragmented nuclei as apoptotic. Necrotic cells were stained with propidium iodide (red) and microglial cells with isolectin B_4_-AlexaFluor488 (green). Scale bars, 100 µm. (**B**) neuronal viability, (**C**) neuronal number and (**D**) microglial number in neuronal-glial co-cultures after incubation with 3 µM tau for 48 h with and without 1 µM YVAD-CHO or 200 nM VX-765. Neuronal viability is expressed as ratio of live and dead (necrotic and apoptotic) cells in a population. Number of neurons and microglia in tau-treated (with/without caspase-1 inhibitors) groups is expressed as the percentage of the total number of appropriate cells in the control (untreated) group, which were considered as 100%. (**E**) interleukin (IL)-1β and -18 level in mixed cell culture medium after incubation with 3 µM tau for 24 h. Data are presented as means ± SE for 5–6 independent experiments; ***p < 0.001 versus untreated control, ^#^p < 0.05, ^###^p < 0.001 versus tau treated cultures.

Neuronal viability and number in neuronal-glial co-cultures were assessed by cell nuclei staining with Hoechst33342 and propidium iodide (PI) (Fig. 2S). Neurons were identified by characteristic morphology in phase contrast images. Cells with homogeneously stained Hoechst33342 (blue) were considered as viable and PI-positive (red) cells as necrotic. Cells with condensed/fragmented nuclei (Hoechst33342, bright blue) were considered as apoptotic. Microglial cells were labelled with isolectin GS-IB_4_ from *Griffonia simplicifolia* and AlexaFluor488 (green) conjugate. Cell cultures were incubated with 4 μg/mL Hoechst33342, 7 μM PI and 7 ng/mL isolectin GS-IB_4_-AlexaFluor488 conjugate for 15 min (5% CO_2_, 37 °C). Cell cultures were analysed under fluorescence microscopy (Olympus IX71S1F-3, USA). The total number of neurons and microglia was counted in 4–5 randomly chosen microscopic fields at 20 × and 10 × magnification, respectively. Quantification was carried out using ImageJ 1.8.0 software.

### Annexin V-Cy3.18 staining

Neuronal-glial co-cultures were washed with PBS several times and incubated with 4.5 μg/ml Annexin V-Cy3.18 conjugate in the incubation buffer (10 mM HEPES, 140 mM NaCl and 2.5 mM CaCl_2_, pH 7.5) for 10 min at room temperature. Cell nuclei were stained with Hoechst33342 (4 µg/mL) and microglial cells were labelled with isolectin GS-IB4- AlexaFluor488 conjugate (7 ng/mL). Cell cultures were washed with incubation buffer and fixed with 4% paraformaldehyde before visualization under fluorescence microscope (Olympus IX71S1F-3, USA). Annexin V-Cy3.18 fluorescence intensity was measured with ImageJ 1.8.0 software. The total number of neurons was counted and Annexin V-Cy3.18 fluorescence intensity was normalized to 100 neurons. For each experimental group at least 4–5 microscopic fields were analysed.

### Evaluation of microglial phagocytic activity

Microglial phagocytic activity was measured using carboxylate-modified 2 μm latex beads. Pure microglial cell cultures were incubated with 0.005% (w/v) latex beads for 2 h (5% CO_2_, 37 °C). Cell nuclei were stained with Hoechst33342 (4 μg/mL) for 5 min. To remove excess beads, the cell cultures were washed with PBS buffer several times and then fixed with 4% paraformaldehyde. Microglial cells were visualised under fluorescence microscope (Olympus IX71S1F-3, Japan) at 40 × magnification. Number of phagocytosed latex beads in microglial cells was calculated using ImageJ 1.8.0 software in at least 5–7 microscopic fields.

### Caspase-1 labelling

Labelling of activated caspase-1was carried out using FamFlica Caspase-1 Assay Kit (ImmunoChemistry Technologies, USA) and provided manufacturer’s protocol. Cell cultures were incubated with FamFlica reagent (1:30 reagent in culture medium) for 1 h. Microglial membrane and cell nuclei were labelled with isolectin GS-IB_4_-AlexaFluor568 conjugate (7 ng/mL) and Hoechst33342 (4 μg/mL). After incubation, cell cultures were washed with wash buffer three times and fixed with 4% paraformaldehyde. Pure microglial cultures were visualized under fluorescence microscopy (Olympus IX71S1F-3, Japan). Analysis of FamFlica fluorescence intensity in pure microglial cultures was performed using ImageJ 1.8.0 software. Fluorescence intensity of FamFlica was normalized to 100 microglial cells. Each experimental group was analysed in 4–5 microscopic fields. Localization of activated caspase-1 in neuron-glial co-cultures was analysed by confocal scanning microscopy (Olympus FV1000, USA).

### Measurement of IL-1β and IL-18

IL-1β and IL-18 level in neuronal-glial co-culture growth medium was measured after 24 h of stimulation with 3 μM tau. After treatments cell culture growth medium was collected and centrifuged at 10,000×*g* for 5 min and then stored at − 20 °C. IL-1β and IL-18 concentration was determined using commercial ELISA kit according to the manufacturer's protocols. Absorbance was measured with Multiscan GO plate reader (Thermo Scientific, USA).

### Statistical analysis

Statistical comparison between independent experimental groups was performed using a one-way ANOVA followed by a Tukey’s test. Statistical analysis was carried out using SigmaPlot (11.0 version software). p values < 0.05 were considered significant. All data are presented as mean ± standard error (SE) of independent cell culture preparations.

### Ethical approval

Experimental procedures involving animals were undertaken in accordance with the EU Directive 2010/63/EU for animal experiments and the Republic of Lithuania law on the care, keeping, and use of experimental animals. Animal care and experimental procedures were approved by Lithuanian State Food and Veterinary Service, ethical approval No. B6(1.9)-855. All experiments were conducted in compliance with the ARRIVE guidelines.

## Results

### Extracellular tau activates caspase-1 within microglial cells via TLR4 receptors and nSMase

**Figure 3:**
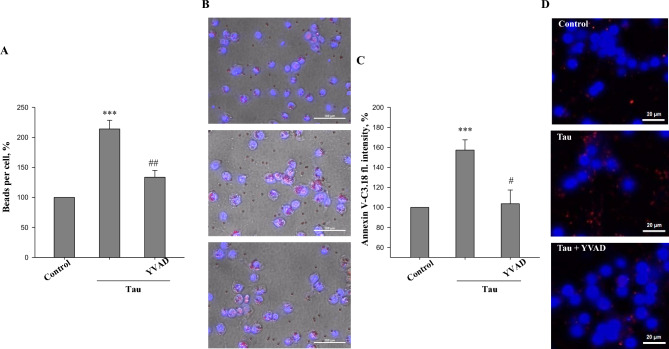
Inhibition of caspase-1 suppresses tau stimulated phosphatidylserine exposure and microglial phagocytic activity. (**A**) phagocytosis of latex beads in pure microglial cell cultures. Changes of latex bead uptake in tau-treated groups (with/without YVAD-CHO) were expressed as percentage of fluorescence intensity in the control (untreated) group, which were considered as 100%. (**B**) representative images demonstrating the uptake of latex beads (red) by microglial cells. (**C**) phosphatidylserine exposure evaluated by Annexin V-Cy3.18 fluorescence intensity in neuronal glial co-cultures. Changes in fluorescence intensity of AnnexinV-Cy3.18 in tau-treated groups (with/wthout YVAD-CHO) were expressed as percentage of fluorescence intensity in the control (untreated) group, which were considered as 100%. (**D**) representative images of phosphatidylserine exposure. Neuronal external phosphatidylserine was labelled with Annexin V-Cy3.18 conjugate (red), cell nuclei was stained with Hoescht33342 (blue). Cell cultures were treated with 3 µM tau for 24 h with or without 1 µM YVAD-CHO. Scale bars, 10 µm. Data are presented as means ± SE for 3 independent experiments; ***p < 0.001 versus untreated control, ^#^p < 0.05, ^##^p < 0.01 versus tau-treated cultures.

Tau protein has been recently demonstrated to activate microglial cells via the NLRP3 inflammasome^26^, which subsequently activates caspase-1^55^. In principle, capase-1 can be activated both in microglial and neuronal cells^62,63^. To determine whether and in which cells tau causes caspase-1 activation, we labelled active caspase-1 in neuronal-glial co-cultures using the FamFlica (FAM-YVAD-FMK FLICA) reagent which covalently binds to the active enzyme and allows to detect caspase-1 inside the cell. As can be seen (Fig. 1A), after 24 h treatment with 3 μM tau protein (2N4R isoform), a FamFlica signal (green colour) co-localized with the microglial marker isolectin-IB_4_ (red); and there was no FamFlica signal in the neuronal cells, which can be seen in the differential interference contrast (DIC) images. Representative images of time-course of caspase-1 activation are provided in Fig. 3S. These data suggest that tau activates caspase-1 in microglial cells.

TLR4 receptors and neutral sphingomyelinase (nSMase) are known to be involved in caspase-1 activation^64,65^. In addition, we have previously shown that nSMase inhibition by GW4869 prevents tau–induced neuronal loss in co-cultures^44^. Therefore, we investigated whether anti-TLR4 antibodies or GW4869 affected tau-induced caspase-1 activation in pure microglia cultures. Results presented in Fig. 1B show that addition of tau protein increased the FamFlica fluorescence intensity by 44 ± 11%, and anti-TLR4 or GW4869 prevented this increase, suggesting that tau-induced caspase-1 activation in microglia is TLR4- and nSMase-dependent.

### Caspase-1 mediates extracellular tau-induced neuronal loss independently of IL-1β and IL-18 secretion

Next we analysed whether the tau–induced neuronal loss could be prevented by caspase-1 inhibitors. Neuronal-glial co-cultures from rat cerebellum were untreated or treated with 3 μM tau and 48 h, later we quantified neuronal viability, neuronal and microglial cell numbers in the cultures (Fig. 2A). Treatment with tau resulted in loss of about 50% of the neurons (Fig. 2C), without any change in the viability of the remaining neurons (Fig. 2B), and a doubling in the number of microglia (Fig. 2D), as previously reported^44^. In these experiments, cell cultures were pre-incubated with two selective inhibitors of caspase-1—Ac-YVAD-CHO (1 μM, for 30 min) and structurally-unrelated VX-765 (200 nM, for 2 h), and then treated with 3 μM tau for 48 h. Both inhibitors completely prevented the tau induced neuronal loss and microglial proliferation, but had no effect on the viability of remaining neurons (Fig. 2). Ac-YVAD-CHO and VX-765 had no direct effect on neuronal viability (Fig. 2B) and number (Fig. 2C) as well on microglial number (Fig. 2D) when were applied without tau protein. This indicates that caspase-1 mediates extracellular tau neurotoxicity in neuronal-glial co-cultures.

**Figure 4:**
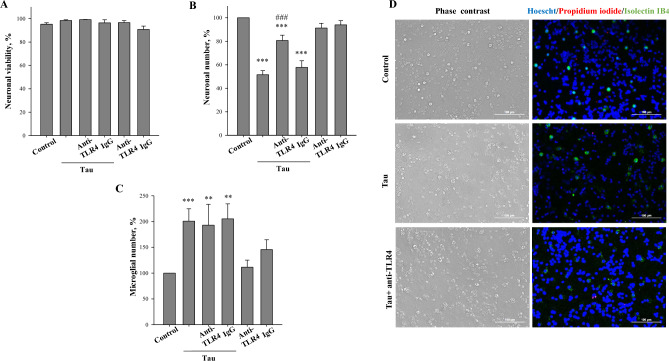
Antibodies blocking TLR4 protect against tau induced neuronal loss. (**A**) neuronal viability, (**B**) neuronal number, (**C**) microglial number. Neuronal viability is expressed as ratio of live and dead (necrotic and apoptotic) cells in a population. Number of neurons and microglia in tau-treated (with/without caspase-1 inhibitors) groups is expressed as the percentage of the total number of appropriate cells in the control (untreated) group, which were considered as 100%. Neuronal-glial co-cultures were incubated with 3 µM tau for 48 h with and without 1 µg/mL anti-TLR4 antibody or 1 µg/ml IgG. In (**A**–**C**) data are presented as means ± SE for 5–6 independent experiments; **p < 0.01, ***p < 0.001 versus untreated control, ^###^p < 0.001 versus tau treated cultures. (**D**) representative images of untreated (control) and tau treated (3 µM for 48 h) neuronal glial-co-cultures with or without 1 µg/mL anti-TLR4 antibody. In phase contrast images, neuronal cell can be identified by characteristic shape and morphology. Cell nuclei were labelled with Hoechst 33342: cells with homogeneously stained nuclei (blue) were considered as viable, cells with condensed or fragmented nuclei as apoptotic. Necrotic cells were stained with propidium iodide (red) and microglial cells with isolectin B_4_-AlexaFluor488 (green). Scale bars, 100 µm.

As caspase-1 can generate mature IL-1β and IL-18, we evaluated whether tau had an effect on IL-1β and IL-18 secretion. We found that the concentration of IL-1β in the growth medium of control neuronal-glial cultures was 44 ± 13 pg/mL and remained unchanged after treatment with 3 μM tau for 24 h—59 ± 19 pg/mL (Fig. 2E). Tau also did not alter the amount of IL-18: the concentration in control and tau-treated groups was 33 ± 3 pg/mL and 27 ± 8 pg/mL, respectively (Fig. 2E). To induce release of IL, we added 3 mM of bzATP (2ʹ(3ʹ)-O-(4-Benzoylbenzoyl)adenosine-5ʹ-triphosphate tri(triethylammonium) salt) to neuronal-glial co-cultures for 1 h. The level of IL-1β in tau-primed cultures after stimulation with bzATP significantly increased up to 303 ± 31 pg/mL (Fig. 4S). Thus, tau protein has no effect on the amount of IL-1β and IL-18 in cell culture medium, suggesting that tau–induced caspase-1 activation and neurotoxicity are not associated with the release of these pro-inflammatory cytokines.

### Caspase-1 mediates tau-induced microglial phagocytosis and exposure of phosphatidylserine on neurons

We have previously shown that tau increases the phagocytic capacity of microglia^44^. Thus, we next investigated whether caspase-1 is involved in stimulation of phagocytic activity of tau-treated microglia using carboxylated beads (mimicking phosphatidylserine-exposing cells). We found that treatment of pure microglia with 3 μM tau for 24 h, increased the uptake of beads from 100% (untreated) to 214 ± 14% (Fig. 3A,B). Pre-treatment with the caspase-1 inhibitor YVAD-CHO strongly reduced the tau-induced increase in microglial phagocytosis.

To be phagocytosed, neurons must expose “eat-me” signals such as phosphatidylserine on the outer surface of plasma membranes^39^. We found that treatment of neuronal-glial co-cultures with tau for 24 h caused phosphatidylserine exposure on neurons as determined by Annexin V-Cy3.18 staining of neurons (Fig. 3C,D): the fluorescence intensity due to Annexin V binding to phosphatidylserine increased by 57 ± 10%, and this was fully prevented when cultures were pre-treated with YVAD-CHO (Fig. 3C). These data indicate that microglial caspase-1 activity is important in causing neuronal phosphatidylserine exposure induced by tau.

### Blockage of TLR4 prevents tau-induced neuronal loss in co-cultures

Various disease-causing proteins, such as Aβ and α-synuclein, are known to activate microglia by binding to TLR4 receptors and thereby stimulate microglia to phagocytose viable neurons^41,48,49^. We tested whether blockage of TLR4 receptors by anti-TLR4 antibodies prevents neuronal loss induced by tau (Fig. 4A–D). Pre-treatment for 30 min with an anti-TLR4 antibody (1 μg/mL) or control immunoglobulin IgG (1 μg/mL), prior to tau exposure for 48 h, had no effect on neuronal viability neither in the absence or presence of tau (Fig. 4A). However, anti-TLR4 antibody partially (59 ± 4%) protected against tau–induced neuronal loss (Fig. 4B), but had no effect on microglia proliferation (Fig. 4C). In contrast, the control IgG had no neuroprotective effect: the numbers of neurons (Fig. 4B) and microglia (Fig. 4C) remained unchanged compared to the tau group. Anti-TLR4 and IgG in the absence of tau had no effect on neuronal viability as well as on numbers of neurons (Fig. 4A,B) and microglia (Fig. 4C). These data suggest that TLR4 receptors mediate extracellular tau-induced neuronal loss but not microglial proliferation.

In this study, we used recombinant tau^2N4R^ expressed in *E. coli* that can result in contamination with bacterial endotoxin (LPS). Bacterial LPS is also known to cause loss of neurons in neuronal-glial co-cultures cultures at 100 ng/ml concentration^42^. However, we have previously shown that at much lower concentrations (which are found in recombinant tau protein preparations) LPS does not exhibit neurotoxic affects in mixed brain cell cultures^44^. These results suggest that tau^2N4R^-induced neuronal loss was not associated with contamination of preparations by LPS.

### The NLRP3 inflammasome mediates tau-induced neurotoxicity

**Figure 5:**
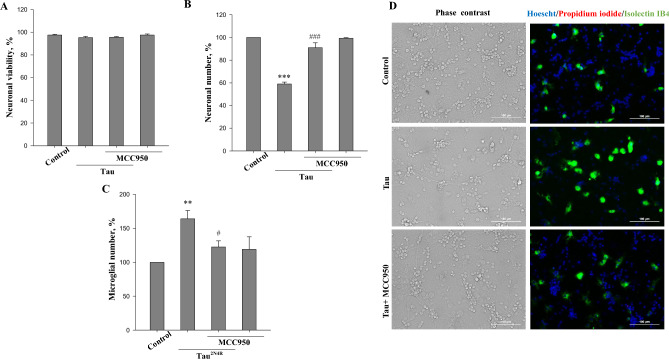
NLPR3 inflammasome inhibitor protects against tau-induced neuronal loss. (**A**) neuronal viability, (**B**) neuronal number, (**C**) microglial number. Neuronal viability is expressed as ratio of live and dead (necrotic and apoptotic) cells in a population. Number of neurons and microglia in tau-treated (with/without NLPR3 inflammasome inhibitor MCC950) groups expressed as the percent of the total number of appropriate cells in the control (untreated) group, which were considered as 100%. Neuronal-glial co-cultures were incubated with 3 µM tau for 48 h with and without 1 µM MCC950. In (**A**–**C**) data are presented as means ± SE for 3 independent experiments; ***p < 0.001, ** p < 0.01 versus untreated control, ^###^p < 0.001, ^#^ p < 0.05 versus tau-treated cultures. (**D**) representative images of untreated (control) and tau treated (3 µM for 48 h) neuronal glial-co-cultures with or without 1 µM MCC950. In phase contrast images, neuronal cell can be identified by characteristic shape and morphology. Cell nuclei were labelled with Hoechst 33342: cells with homogeneously stained nuclei (blue) were considered as viable, cells with condensed or fragmented nuclei as apoptotic. Necrotic cells were stained with propidium iodide (red) and microglial cells with isolectin B_4_-AlexaFluor488 (green). Scale bars, 100 µm.

Caspase-1 can become activated by the NLRP3 inflammasome^55^, which was shown to regulate tau-seeding^26^ and brain atrophy in P301S tauopathy models^57^. Thus, we evaluated whether the NLRP3 inflammasome inhibitor MCC950 has an effect on extracellular tau neurotoxicity (Fig. 5A–D). We found that after pre-incubation of neuronal-glial co-cultures with 1 µM MCC950, tau did not alter neuronal viability (Fig. 5A) and their numbers (Fig. 5B) compared to cultures not treated with tau. Additionally, tau-stimulated microglial proliferation was substantially reduced (Fig. 5C). MCC950 alone had no effect on neurons (Fig. 5A,B) and microglia (Fig. 5C). Thus, the NLRP3 inflammasome appears to mediate tau-induced microglial activation and neuronal loss.

### NADPH oxidase is involved in tau-induced neuronal loss

**Figure 6:**
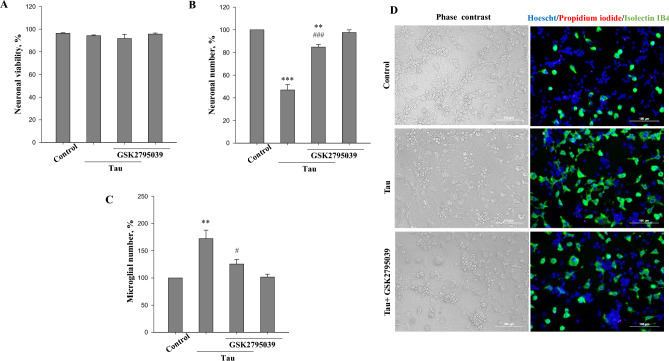
NADPH oxidase inhibitor supresses tau-induced neurotoxicity. (**A**) neuronal viability, (**B**) neuronal number, (**C**) microglial number. Neuronal viability is expressed as ratio of live and dead (necrotic and apoptotic) cells in a population. Number of neurons and microglia in tau-treated (with/without NADPH oxidase inhibitor GSK2795039) groups expressed as the percentage of the total number of appropriate cells in the control (untreated) group, which were considered as 100%. Neuronal-glial co-cultures were incubated with 3 µM tau for 48 h with and without 1 µM GSK2795039. In A, B and C data are presented as means ± SE for 3–5 independent experiments; **p < 0.01, ***p < 0.001 versus untreated control, ^#^p < 0.05, ^###^p < 0.001 versus tau-treated cultures. (**D**) representative images of untreated (control) and tau treated (3 µM for 48 h) neuronal glial-co-cultures with or without 1 µM GSK2795039. In phase contrast images, neuronal cell can be identified by characteristic shape and morphology. Cell nuclei were labelled with Hoechst 33342: cells with homogeneously stained nuclei (blue) were considered as viable, cells with condensed or fragmented nuclei as apoptotic. Necrotic cells were stained with propidium iodide (red) and microglial cells with isolectin B_4_-AlexaFluor488 (green). Scale bars, 100 µm.

TLR4 can activate NOX to produce ROS^58,59^. We evaluated whether pre-incubation of neuronal-glial co-cultures with the NOX2 inhibitor GSK2795039 (1 µM for 30 min) protects against tau neurotoxicity (Fig. 6A–D). As shown in Fig. 6A and B, GSK2795039 largely prevented tau-induced neuronal loss without affecting neuronal viability. We also found that GSK2795039 reduced the tau-induced microglial proliferation (Fig. 6C). GSK2795039 in the absence of tau had no effect on neurons (Fig. 6A,B) and microglia (Fig. 6C).These data suggest that NOX mediates tau–induced microglial activation and neurotoxicity in neuronal-glial co-cultures.

## Discussion

A growing body of evidence suggests that, in AD patients and tau-transgenic mice, cognitive deficits and neuronal loss correlate with tau pathology^66–71^. However, the mechanisms by which tau protein causes neuronal loss are as yet unclear. We have previously shown that extracellular tau protein induced phagocytosis of stressed-but-viable neurons^44^ and here we show that tau acts via caspase-1 activation. During exposure to extracellular tau, caspase-1 becomes activated in microglial cells, and caspase-1 inhibitors entirely prevent neuronal loss in neuronal-glial co-cultures. In addition, we found that caspase-1 was activated via TLR4, and blocking TLR4 with an antibody suppresses tau induced neuronal loss. Although our findings indicated that TLR4 inhibition provides neuroprotection against extracellular tau-induced neuronal loss, others have shown that mild stimulation of TLR4 by LPS reduced level of intracellular phospho-tau and improved cognitive functions via activation of autophagy in tau P301S transgenic mice^72^. Thus, TLR4 may have multiple roles in tau-associated pathology. To the best of our knowledge, currently there is no data on how tau protein activates TLR4 receptor mediated signalling—by direct binding or through receptor cross-talk. Recently we have shown that extracellular tau protein induced neurotoxicity is also abolished by pharmacological and genetic ablation of P2Y6 receptors^46^, thus suggesting that multiple membrane surface receptors can be involved in tau-induced pathology. Microglial pro-inflammatory receptors inhibition is considered as one of strategies for treatment of neurodegenerative diseases^73^. Therefore it is important to elucidate precise mechanisms of tau interactions with microglial receptors in further studies.

Activation of TLR4 receptors promotes the priming of the NLRP3 inflammasome, which catalyses the conversion of pro-caspase-1 to caspase-1^64^. In our study, an inhibitor of the NLRP3 inflammasome prevented tau-induced neuronal loss suggesting that the NLRP3 inflammasome-caspase-1 pathway is involved in extracellular tau-induced microglial neurotoxicity. In general, inflammasome assembly and subsequent caspase-1 activation is multistep process initiated by triggering of cell surface receptors (e.g. TLR receptors, purinergic receptors P2X7) by their ligands and cell metabolism disruption^74^. NLRP3 inflammasome assembly is known to be promoted by excessive ceramide and ROS production^64,75^. Previously we have shown that the nSMase inhibitor (GW4869) protects against tau-neurotoxocity^44^ and in this study GW4869 was able to block caspase-1 activation, implying nSMase is involved in inflammasome/caspase-1 activation. Overall, since (i) suppression of the NLRP3 inflammasome reduced hippocampal atrophy in P301S tau mice^57^, (ii) knockout of the microglial phagocytic receptor P2Y6 prevented neuronal loss in P301S tau mice^46^, and (iii) activated microglia were shown to phagocytose stressed-but-viable neurons containing P301S-tau inclusions in vitro^45^, we can speculate that NLRP3 inflammasome activation in microglial cells might be required for both extracellular and intracellular tau-induced neuronal death by phagocytosis. However, this requires further investigation. Interestingly, in contrast to Jiang et al. despite activation of caspase-1 we did not observe any changes in IL-1β and IL-18 levels in the cell culture medium after stimulation of neuronal-glial co-cultures with exogenous tau protein^76^. On the other hand, caspase-1 activation and IL-1β secretion were shown to be uncoupled events^77^ and inflamamsome-dependent IL-1β secretion has been found to depend on membrane permeabilisation^78^. Moreover, IL-1β processing-independent neuroprotective effects of caspase-1 inhibition were also reported in the models of neuroAIDS^79^, amyotrophic lateral sclerosis^80^ and oxygen-and-glucose deprivation-induced neuronal cell death^81,82^. Here we show that caspase-1 inhibition prevented tau-induced microglial phagocytosis and the consequent loss of neurons.

**Figure 7:**
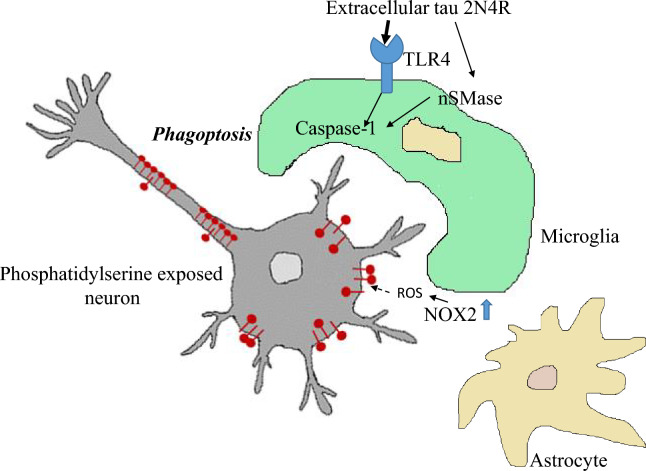
Possible mechanism of tau-induced phagoptosis. Tau protein activates microglial TLR4 receptors and neutral sphingomyelinase (nSMase) which in turn induce caspase-1 and NADPH oxidase 2 (NOX2) activation, which leads to reactive oxygen production (ROS). ROS may lead to phosphatidylserine exposure on neurons. This can be recognized by microglia triggering microglial phagocytosis of stressed-but-viable neurons.

NOX is a membrane-bound multi-subunit enzyme generating ROS in response to a wide variety of stimuli^83^. We found that inhibition of NOX2 partly prevents tau induced neuronal loss in neuronal-glial co-cultures suggesting that NOX2 produced ROS may be involved in phagoptosis of neurons. We have previously shown that tau induces glial cell dependent exposure of phosphatidylserine on neurons^44^. Thus, we can speculate that extracellular tau activates NOX in glial cells resulting in ROS production that causes phosphatidylserine exposure on outer leaflets of membranes of nearby neurons leading to microglial phagocytosis of affected viable neurons (Fig. 7). However, NOX2 is known to be expressed in glial cells (microglia and astrocytes) as well as in neurons^84^, thus we cannot exclude the possibility that NOX2 was activated in several brain cell types. These and our previously published results^44^ indicate that extracellular tau neurotoxicity is not associated with TNFα, NO, IL-1β or IL-18 production. Thus, NOX and ROS formation could be the critical mechanism of microglia-mediated neuronal loss induced by extracellular tau. Importantly, it has been reported that knockdown of NOX4 protected against accumulation of pathological tau and suppressed cognitive decline in a mouse model of (P301L tau) tauopathy^85^. This suggest that NOX activation mediates both extracellular and intracellular tau protein pathology.

Overall, our results suggest that extracellular tau stimulates microglial cells to phagocytose stressed-but-viable neurons via activation of TLR4 receptors, NLRP3 inflammasome, caspase-1 and NOX (a possible mechanism is provided in Fig. 7). Importantly, NLRP3 inflammasome and caspase-1 have been shown to be activated in mild cognitive impairment and AD brains^52,86^. Although there is considerable evidence that Aβ can induce NLRP3 and caspase-1-associated neuroinflammation^49,87,88^, recent studies have shown that this inflammatory pathway is also upregulated in patients with primary tauopathy (FTD) and Tau22 transgenic mice, suggesting that NLRP inflammasome and caspase-1 activation is directly related to pathological changes of tau protein^56^. Moreover, NOX4 was also shown to be activated in AD and FTLD brain^85^. Thus, the NLRP3 inflammasome, caspase-1 and the NOX may be potential targets for treatment of AD and primary tauopathies.

## Conclusion

Toll-like 4 receptor–NLRP3 inflammasome–caspase-1 signalling axis mediates extracellular tau protein-induced microglial phagocytosis of stressed-but-viable neurons.

## Supplementary Information

Supplementary Figures.

## Data Availability

The data that support the findings of this study are available from the corresponding author upon request.

## Abbreviations

AD: Alzheimer’s disease
Aβ: β-Amyloid
bzATP: 2ʹ(3ʹ)-O-(4-Benzoylbenzoyl)adenosine-5ʹ-triphosphate tri(triethylammonium) salt
CD33: Sialic acid binding immunoglobulin-like lectin 3
CR1: Complement receptor 1
DIC: Differential interference contrast
FBS: Fetal bovine serum
FTD: Frontotemporal dementia
HBSS: Hank's balanced salt solution
HEPES: 4-(2-Hydroxyethyl)-1-piperazineethanesulfonic acid
IgG: Immunoglobulin G
IL: Interleukin
LPS: Lipopolysaccharide
MW: Molecular weight
NOX: NADPH oxidase
NLRP3: Leucine-rich repeat-containing protein-3
nSMase: Neutral sphingomyelinase
P/S: Penicillin/streptomycin
PAPM: Pathogen-associated molecular pattern
PBS: Phosphate-buffered saline
PI: Propidium iodide
PLL: Poly-(l)-lysine
ROS: Reactive oxygen species
SE: Standard error
Tau: Tubulin associated unit
TLR4: Toll-like 4 receptor
TNF-α: Tumor necrosis factor α
TREM2: Triggering receptor expressed on myeloid cells 2
UDP: Uridyl diphosphate
