# Supplementary material for: Extracellular tau stimulates phagocytosis of living neurons by activated microglia via Toll-like 4 receptor–NLRP3 inflammasome–caspase-1 signalling axis
Source: Sci Rep. 2023 Jul 4;13:10813. doi: 10.1038/s41598-023-37887-3 (PMC10319744; doi:10.1038/s41598-023-37887-3)
Supplement: Supplementary file 1 — Supplementary Figures. [file 41598_2023_37887_MOESM1_ESM.docx]

**Supplementary material**

Katryna Pampuscenko, Ramune Morkuniene, Lukas Krasauskas, Vytautas Smirnovas, Guy C. Brown, Vilmante Borutaite. **Extracellular tau stimulates phagocytosis of living neurons by activated microglia via Toll-like 4 receptor – NLRP3 inflammasome – caspase-1 signalling axis**

**
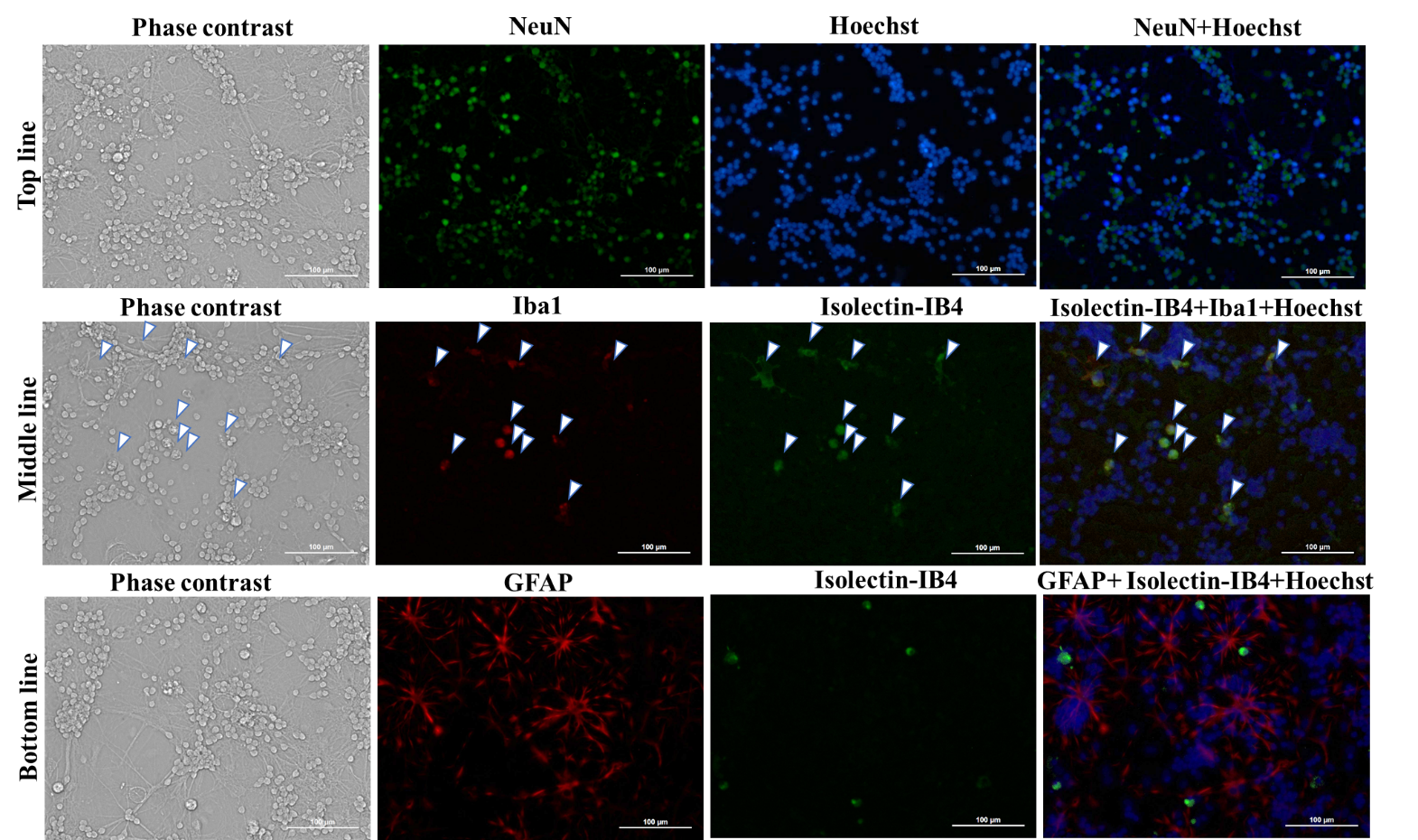
Representative images of composition of neuronal-glial co-cultures**

**Figure 1S. Representative images of neuronal-glial co-cultures from rat cerebellum.** Neuronal-glial co-cultures consist of neurons, microglia and astrocytes. Top line: neurons can be identified according to characteristic shape and morphology, and they are positive for NeuN, which is a marker of postmitotic neurons. Middle line: microglial cells (white arrow) are Iba-1 positive, and Iba-1 staining co-localize with isolectin GS-IB4 stain. Bottom line: astrocytes are GFAP positive cells.

Cell cultures were fixed with with 4 % paraformaldehyde (15 min) and then blocked with using 0.1 M glycine (5 min). Non-specific interactions were blocked with 10 % BSA (1 h). Cell cultures then were incubated with 1:200 anti-GFAP, anti-NeuN and Iba-1 antibodies overnight (4 ^o^C) followed by further incubation with 1:200 AlexaFuor555 (for anti-GFAP and anti-Iba1) or AlexaFuor448 (for anti-NeuN) secondary antibody for 2 h. Microglia was additionally labeled with isolectin GS-IB4 from *Griffonia simplicifolia* conjugated with AlexaFluor488 at 15 µg/ml concentration and cell nuclei were labelled with 7 ng /ml Hoechst 33342. Pictures were taken with fluorescence microscope (Olympus IX71S1F-3, USA). Scale bars, 100 μm

**Representative images of cell viability and density assessment by fluorescence microscopy**

**
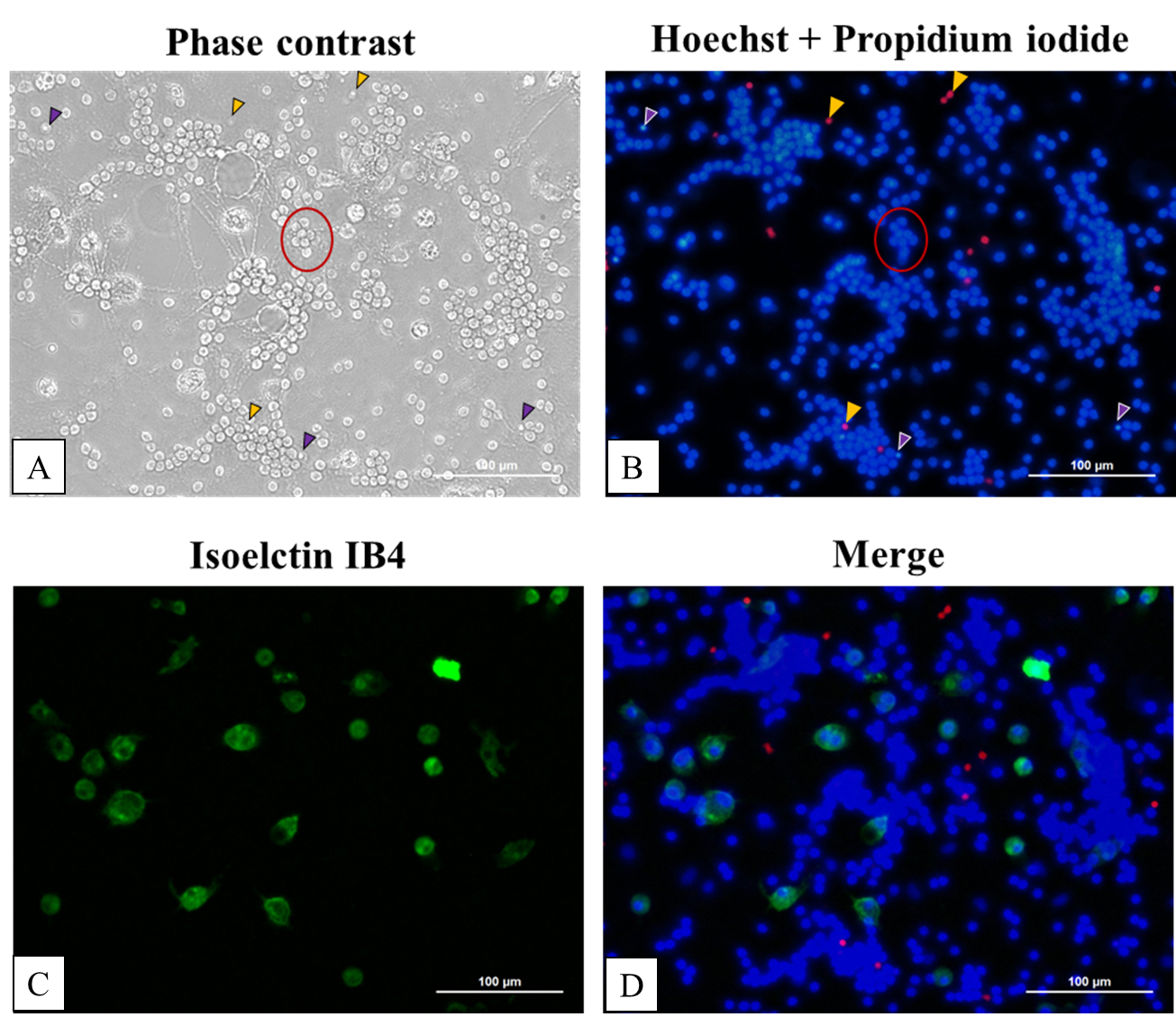
**

**Figure 2S. Representative images of cell viability and density assessment in neuronal-glial co-cultures.** Neuronal viability and number in neuronal-glial co-cultures were assessed by cell nuclei staining with Hoechst33342 and propidium iodide (PI). Neurons were identified by characteristic morphology in phase contrast images (A, red circle). Cells with homogeneously stained Hoechst33342 (blue) were considered as viable (A and B, red circle) and PI-positive (red) cells as necrotic (A and B, yellow arrows). Cells with condensed/fragmented nuclei (Hoechst33342, bright blue) were considered as apoptotic (A and B, violet arrows). Microglial cells were labelled with isolectin GS-IB4 from *Griffonia simplicifolia* and AlexaFluor488 conjugate (C, green cells). Cell cultures were incubated with 4 μg/ml Hoechst33342, 7 μM PI and 7 ng/ml isolectin GS-IB4-AlexaFluor488 conjugate for 15 min (5% CO_2_, 37 ^o^C). Cell cultures were analysed under fluorescence microscopy (Olympus IX71S1F-3, USA).

**Representative images of tau protein-induced caspase-1 activation in neuronal-glial co-cultures**


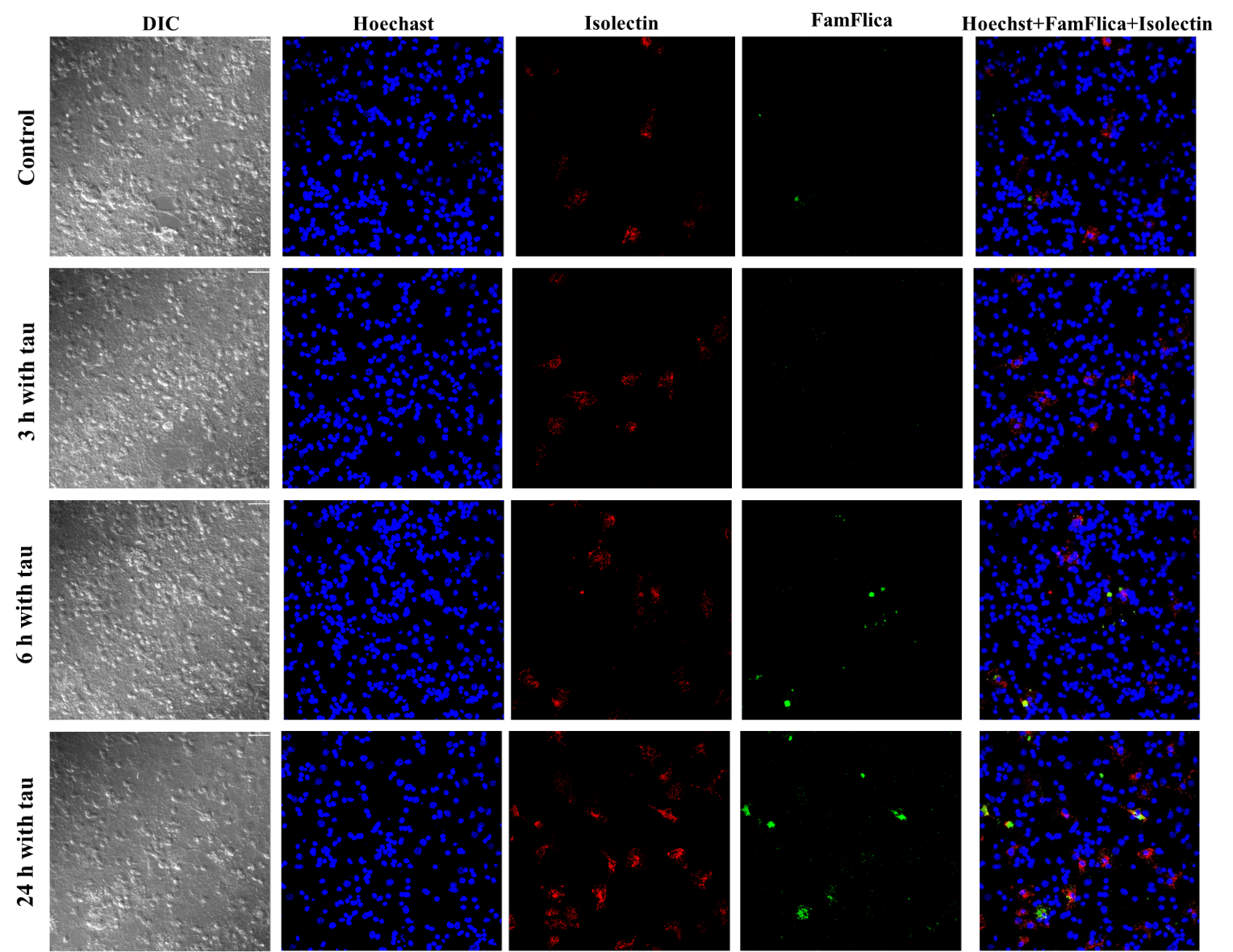


**Figure 3S. Representative images of time-course of caspase-1 activation in neuronal-glial co-cultures induced by extracellular tau protein.**

Neuronal-glial co-cultures were treated with 3 μM tau protein (2N4R isoform) for 3 h, 6 h and 24 h. There was negligible FamFlica signal in control (untreated) group and in cell cultures which ere incubated with tau for 3 h. Meanwhile, after 6 h treatment with tau protein (2N4R isoform), a FamFlica signal (green colour) co-localized with the microglial marker isolectin-IB4 (red); and the signal aggravated after 24 h of treatment. There was no FamFlica signal in the neuronal cells, which can be seen in the differential interference contrast (DIC) images.

Activated caspase-1was labelled using FamFlica Caspase-1 Assay Kit (ImmunoChemistry Technologies, USA) according to manufacturer's protocol. In brief, cell cultures were incubated with FamFlica reagent (1:30 reagent in culture medium) for 1 h and then were washed with wash buffer three times. Microglial membrane and cell nuclei were labelled with isolectin GS-IB4-AlexaFluor568 conjugate (7 ng/ml) and Hoechst33342 (4 μg/ml). Cell cultures were fixed with 4% paraformaldehyde and were visualized under confocal scanning microscopy (Olympus FV1000, USA). Scale bars, 10 µm.

**Figure 4S. Level of interleukin-1β in neuronal-glial co-culture cell medium**

Neuronal-glial co-cultures were treated with 3 μM tau protein (2N4R isoform) for 24 h. Where indicated, after treatment with tau, 3 mM bzATP (2ʹ(3ʹ)-O-(4-Benzoylbenzoyl)adenosine-­5ʹ-triphosphate tri(triethylammonium) salt,) was added for 1 h. After treatments cell culture growth medium was collected and centrifuged at 10000×g for 5 min and then stored at -20^o^C. IL-1β concentration was determined using commercial ELISA kit (Abbexa, UK) according to the manufacturer's protocol. The level of IL-1β in control, tau and bzATP groups were below the detection limit (<31.2 pg/ml). Stimulation of tau-primed cells with 3 mM bzATP for 1 h resulted in detectable concentrations (303±31 pg/ml) of IL-1β in cell culture medium supernatants.
